# Poly-L-Arginine Molecule Properties in Simple Electrolytes: Molecular Dynamic Modeling and Experiments

**DOI:** 10.3390/ijerph19063588

**Published:** 2022-03-17

**Authors:** Maria Morga, Piotr Batys, Dominik Kosior, Piotr Bonarek, Zbigniew Adamczyk

**Affiliations:** 1 Jerzy Haber Institute of Catalysis and Surface Chemistry, Polish Academy of Sciences, Niezapominajek 8, PL-30239 Krakow, Poland; piotr.batys@ikifp.edu.pl (P.B.); dominik.kosior@ikifp.edu.pl (D.K.); 2 Department of Physical Biochemistry, Faculty of Biochemistry, Biophysics and Biotechnology, Jagiellonian University, Gronostajowa 7, PL-30387 Krakow, Poland; piotr.bonarek@uj.edu.pl

**Keywords:** conformations of poly-L-arginine, density of poly-L-arginine, electrokinetic charge of poly-L-arginine molecule, hydrodynamic diameter of poly-L-arginine, molecular dynamics modeling, poly-L-arginine solutions, viscosity of poly-L-arginine, poly-L-arginine molecule conformations and structure

## Abstract

Physicochemical properties of poly-L-arginine (P-Arg) molecules in NaCl solutions were determined by molecular dynamics (MD) modeling and various experimental techniques. Primarily, the molecule conformations, the monomer length and the chain diameter were theoretically calculated. These results were used to interpret experimental data, which comprised the molecule secondary structure, the diffusion coefficient, the hydrodynamic diameter and the electrophoretic mobility determined at various ionic strengths and pHs. Using these data, the electrokinetic charge and the effective ionization degree of P-Arg molecules were determined. In addition, the dynamic viscosity measurements for dilute P-Arg solutions enabledto determine the molecule intrinsic viscosity, which was equal to 500 and 90 for ionic strength of 10^−5^ and 0.15 M, respectively. This confirmed that P-Arg molecules assumed extended conformations and approached the slender body limit at the low range of ionic strength. The experimental data were also used to determine the molecule length and the chain diameter, which agreed with theoretical predictions. Exploiting these results, a robust method for determining the molar mass of P-Arg samples, the hydrodynamic diameter, the radius of gyration and the sedimentation coefficient was proposed.

## 1. Introduction

Macroions, due to their effective adsorption at various surfaces, are widely used in biotechnology and in medicine to enhance antibacterial and antimicrobial activity [1,2,3,4,5,6,7]. In other processes macroion adsorption is applied for modification of substrates for protein and enzyme immobilization [8], separation and biosensing processes [9]. Additionally, consecutive adsorption of cationic and anionic macroions using the layer-by-layer (LBL) technique has been effectively applied in nanocapsule formulation for RNA and DNA vaccines and drug delivery [10,11,12,13,14,15].

Among various macroions, poly-L-arginine (P-Arg) has attracted special interest due to its unique properties and its environmentally friendly and biocompatible behavior [13,16]. It represents a cationic biopolymer composed of physiologically active L-arginine amino acid.

On a molecular level, L-arginine contains a positively charged α-amino group, an α-carboxylic acid group and a 3-carbon aliphatic straight chain ending with a guanidine group, constituting the side chain of the monomer structure [17]. Under physiological pH, the carboxylic acid is deprotonated (−COO^−^) (pKa > 2.2) [18], the α-amino group is protonated (−NH_3_^+^) (pKa > 9.0) [17,18] and the guanidine group is also protonated resulting in the creation of the guanidinium form (-C-(NH_2_)_2_^+^), which makes the poly-L-arginine aliphatic amino acid charged even under alkaline conditions (pKa > 12.5) [17,18,19,20].

Because of its unique properties, P-Arg has been extensively studied as a drug nanocarrier [13,16], as a component of gene delivery systems [10,11], in the build-up of multilayers for wound healing dressings [21] and as an antibacterial and antimicrobial agent [16] as well as in anticancer vaccines [22], anticancer immunotherapy [23], RNA delivery [12,24], protein immobilization [25] and biosensors [26,27].

Considering delivery systems, P-Arg is widely studied as a polypeptide-type gene carrier with the ability to fold genomic DNA and with high transfection efficiency [10,28]. Since the nucleic acids in their free form are rapidly degraded by nucleases active in extracellular matrices, native forms of nucleic acids are characterized by poor pharmacokinetics [28]. The intercellular traffic steps are known to be based on charge density, structure of the peptides and PKa values [10]. The transcription efficiency is suppressed at large molar mass (MW) and correlates with the polyplex forming ability expressed as a critical ratio of the number of polypeptide cationic groups to the number of pDNA anionic groups [10]. In this respect the basic research on peptide structural changes, effective electrokinetic charge and the exact value of the MW contributes to the development of new methods of obtaining matrices based on the polypeptide core [10,28,29,30]. Gene therapies based on the application of nucleic acids are of significant interest considering the treatment of diseases of a genetic ethology and innovative vaccine formulations.

Because of the wide range of applications, extensive research has been conducted in order to investigate P-Arg structure [31], surface properties [18,32] and adsorption at various substrates [25,33,34].

Opanasopit et al. [34] investigated the application of P-Arg-coated liposomes for gen delivery. Such cationic complexes are hypothesized to deliver DNA through the endosomal pathway. The advantage of using P-Arg is that cationic polymer vectors provide flexible DNA-carrying capacity and are simple to use.

In Reference [21] the formation of a multilayer composed of fucoidan and P-Arg was studied. The work focused on formation and enzymatic breakdown of the macroions for a potential surface treatment for wound dressing. In this study, P-Arg was used as a component of the polyelectrolyte film intended as a NO donor after being degraded by trypsin to arginine. P-Arg was paired with fucoidan, a polysaccharide known from its antimicrobial/anti-inflammatory properties. The film build-up was studied using the quartz crystal microbalance (QCM). The zeta potential was also determined upon the film build-up revealing the reversal of the zeta potential upon the adsorption of each subsequent polycation.

In the work of Li et al. [20] P-Arg has been extensively studied as an antibacterial agent. Due to the presence of the guanidinium group, responsible for the positive charge on P-Arg-coated metal nanoparticles and its hydrophobic character, the electrostatic interactions between bacterial cells and the nanoparticles and the solvation of the nanoparticles into hydrophobic membrane are strengthen, thus the antibacterial action is enhanced.

Although P-Arg application efficiency depends on their molar mass [34,35], specific density, size [35], geometry (conformation) and their surface charge [35], few investigations have been carried out focusing on thorough physicochemical characteristics of P-Arg molecules in electrolyte solutions, especially determining the molecule structure, conformations, surface charge and ionization degree. The lack of such fundamental data prohibits a quantitative interpretation of experimental results concerning P-Arg adsorption in solid substrates, multilayer formation efficiency, incorporation of P-Arg molecules in the liposome structure or their interactions with cell membranes.

Therefore, the objective of this work was to perform thorough experimental investigation of P-Arg solutions under various ionic strengths and pHs using complementary experimental techniques. This allowed the diffusion coefficients, the hydrodynamic diameter of the P-Arg molecule, its electrophoretic mobility, the effective (electrokinetic) charge and the intrinsic viscosity to be determined. The experimental data were quantitatively interpreted in terms of all-atom molecular dynamics (MD) modeling. As a result, P-Arg molecule conformations, especially the molecule chain diameter, extended length and effective cross-section area, were quantitatively determined for the first time. Exploiting these results, a robust method for determining the molar mass of P-Arg samples, the molecule hydrodynamic diameter, the radius of gyration and the sedimentation coefficient based on the viscosity measurements was developed.

In addition, the acquired results can be exploited -for controlling P-Arg molecule adsorption at solid/liquid interfaces and for a quantitative interpretation of experimental data obtained from quartz microbalance [36,37], reflectometry, ellipsometry, streaming potential measurements [38,39] and other experimental techniques that require proper calibration.

## 2. Experimental Section

### 2.1. Materials and Methods

Poly-L-arginine hydrochloride (P-Arg), a synthetic polyamino acid with an average molar mass of 42 kg mol^−1^ (determined by manufacturer), hereafter referred to as P-Arg, was purchased from Sigma Aldrich Merck KGaA, Darmstadt, Germany.

The P-Arg solutions were prepared by dissolving a proper mass of dried P-Arg crystalline powder in a proper solvent (water or NaCl solutions) of fixed pH and ionic strength.

The NaCl solutions of desired ionic strengths and pH were prepared using deionized water obtained from a Milli-Q Elix & Simplicity 185 purification system, Millipore SAS Molsheim, France, and analytical grade NaCl was purchased from Sigma Aldrich Merck KGaA, Darmstadt, Germany.

The HCl and NaOH were used for fixing pH of the samples and were also products of Sigma Aldrich. All reagents were analytical grade and used within the experiments without further purification.

The bulk properties comprising electrophoretic mobility and diffusion coefficients of P-Arg molecules at various conditions were determined by electrophoretic measurements (Laser Doppler Velocimetry (LDV) technique) and dynamic light scattering (DLS) using the Malvern Zetasizer Nano ZS apparatus.

The CD spectra were recorded at 293 K on a JASCO J-710 spectropolarimeter. A cylindrical quartz cuvette with a 200 μm path length was used. Due to the polydispersity of the PARG, the spectra were normalized by the peptide bond concentration of 1.82 mM. Three scanning acquisitions were collected and averaged to yield the final spectrum, which was then corrected by the solution baseline. The secondary structure composition was determined using the BeStSel web server [40,41]. Error estimates are based on the standard deviation of the data.

The density of P-Arg solutions under various ionic strengths was determined using the high precision Anton Paar DMA 5000 M densitometer.

The dynamic viscosity of P-Arg solutions of defined mass concentrations was measured using a certified Cannon–Ubbelohde semi-micro dilution viscometer, which requires small volumes of polypeptide solution (below 5 mL) and permits easy serial dilutions. The whole set-up was equipped with a thermostat that allowed precise control of temperature during the measurement. For the extrapolation to infinite dilution limit, up to 10 data points were provided under the experimental range of 50 to 500 mg L^−1^ of P-Arg mass concentration at given ionic strengths, where the relative viscosity *η_r_* was lower than 1.2. The precision of intrinsic viscosity determination was ca. 1%. All measurements were performed at 298 K.

### 2.2. Theoretical Modeling

GROMACS 5.1.4 package [42,43] was used for all-atom detail molecular dynamics (MD) modeling of the fully charged P-Arg molecule. The simulation protocol was based on Reference [44]. The Amber03 force field [45] was applied to describe the peptide, while the explicit TIP3P model was employed for water [46]. The P-Arg molecules with 25, 30, 35, 40, 45 and 50 repeat units were generated using Avogadro software [47]. Obtained structures were solvated and the Na^+^ and Cl^−^ ions were added to neutralize the systems and set the ionic strength equal to 10^−3^ M. The modeling was carried out by applying periodic boundary conditions and in NPT ensemble. Prior to the 150 ns long production run, energy minimization was performed. The first 50 ns of the production run was disregarded in the analysis as the relaxation time. A V-rescale thermostat [48] and an isotropic Parrinello-Rahman barostat [49] were used to control the temperature and pressure (*T* = 298 K, *p* = 1 bar). The coupling constants were 0.1 and 2 ps, respectively. All the bonds in the macroions and water molecules were controlled by the LINCS [50] and SETTLE [51] algorithms, respectively. A 2 fs timestep within the leap-frog integration scheme was applied, and the trajectories were saved every 10 ps. The VMD software package was used for visualization [52].

The density of P-Arg was determined computationally via the dilution method presented in References [44,53]. In brief, the size of simulation boxes containing the P-Arg molecule were reduced systematically, which increased the molecule mass fraction (*w_p_*) from 9.7 × 10^−4^ to 1.2 × 10^−2^. The densities of the systems (*ρ*_sys_) and pure solvent (*ρ*_sol_) were used to calculate the specific density of the solute (*ρ*_s_) from the following dependence: $\rho_{s}=\rho_{\mathrm{sol}}/1+s_{p}$ [44,53,54].

## 3. Results and Discussion

### 3.1. Theoretical Modeling Results

The MD simulations performed for P-Arg*_N_*_m_ molecules, composed of a various number of repeating units *N*_m_, provide the density of the molecule in aqueous solutions, its conformations comprising the maximum extended (contour) length and the chain diameter. Obtained results can be extrapolated to larger molar mass, as previously shown for poly-L-lysine [44].

**Figure 1:**
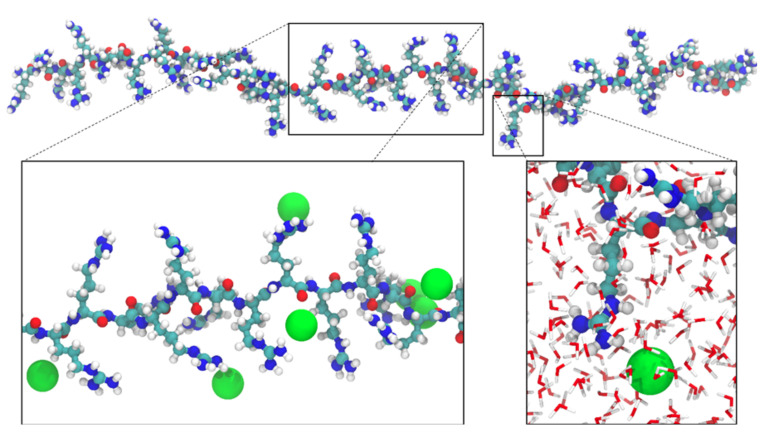
Snapshot of the P-Arg_50_ molecule derived from MD modeling at 10^−3^ M NaCl. The Cl^−^ ions are shown as green spheres characterized by the van der Waals radius. The N, O, C and H atoms are highlighted in blue, red, cyan and white, respectively.

Typical P-Arg_50_ molecule conformation obtained for the ionic strength of 10^−3^ M NaCl is shown in Figure 1. Despite the low ion concentration, significant counterion condensation is visible, as in the case of other strongly charged polypeptides [44].

Primarily, in the MD modeling the P-Arg molecule density was determined via the dilution method as described in Reference [53]. The obtained dependence of *ρ_sol_/ρ_sys_* on *w_p_* determined by MD is shown in Figure S1. Thus, *ρ_p_* = 1.58 ± 0.03 × 10^3^ kg m^−3^ is obtained (Table 1). Using this value one can calculate that the volume of a single monomer 

*ν_1_ = M*_1_*/(A_v_ ρ_p_)* is equal to 0.183 ± 0.002 nm^3^, where *M_1_* is the monomer molar mass equal to 0.174 kg mol^−1^ and Av is the Avogadro constant.

The physicochemical parameters determined for the P-Arg molecules from MD modeling are gathered in Table 1.

**Figure 2:**
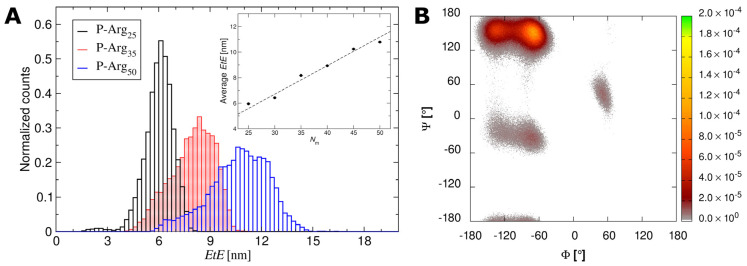
(**A**) The end-to-end distance distribution for *N_m_* = 25, 35 and 50 derived from MD modeling (NaCl concentration equal to 10^−3^ M). The inset shows the average P-Arg end-to-end (*EtE*) length as a function of the number of monomers. The dashed line denotes linear fitting—using Equation (1). (**B**) Ramachandran plot for P-Arg_50_ calculated from the molecular dynamics simulations showing the distribution of the Ψ and Φ angles of the peptide backbone.

In further calculations, much attention was devoted to the calculations of the end-to-end (EtE) length of the molecule considering that in every MD run, conformations of the molecule are fluctuating in time. In order to quantify this effect, histograms presenting EtE length of the molecule for various monomer numbers were produced and are shown in Figure 2A for P-Arg molecule composed of 25, 35 and 50 monomers (NaCl concentration of 10^−3^ M). One can observe that the average EtE length can be approximated by a linear function of the number of monomers *N_m_* (see Figure 2A inset).

(1) $$L_{e}=l_{a}N_{m}$$

where *l_a_* is the constant equal to 0.225 ± 0.001. It should be mentioned that using 

Equation (1) one can predict by extrapolation the average length of the P-Arg molecule of much larger molar mass than is feasible in MD modeling. In addition, from such histograms the average and the maximum EtE distance corresponding to the molecule contour length at low ionic strength limit can be determined; see Table 2.

The average, the maximum EtE distance (corresponding to the molecule contour length at low ionic strength limit) and other parameters derived from the MD modeling are shown in Table 2. The chain diameters calculated from simulations of P-Arg molecule with different number of repeat units are consistent. Therefore, the simulations of relatively short molecules proved to be sufficient for the production of the properties of molecules of larger molar mass.

In Figure 2B, Ramachandran plots calculated based on the MD simulation trajectories show the secondary structure distribution of the P-Arg_50_ molecule. The top-left spots correspond to backbone configurations characteristic of β-sheets, while the center-left and the center-right spots correspond to α-helices [55]. Performed analysis confirms visual inspection (see Figure 1) that the chain is extended and partially forms a single strand β-sheet conformation. Additionally, a very small α-helical contribution was noticed. The similar features were observed for the shorter P-Arg chains.

### 3.2. Experimental Characteristics of P-Arg Solutions

Initially, the secondary structure of the P-Arg molecules in NaCl solutions was investigated using circular dichroism (CD) performed according to the above described procedure. 

**Figure 3:**
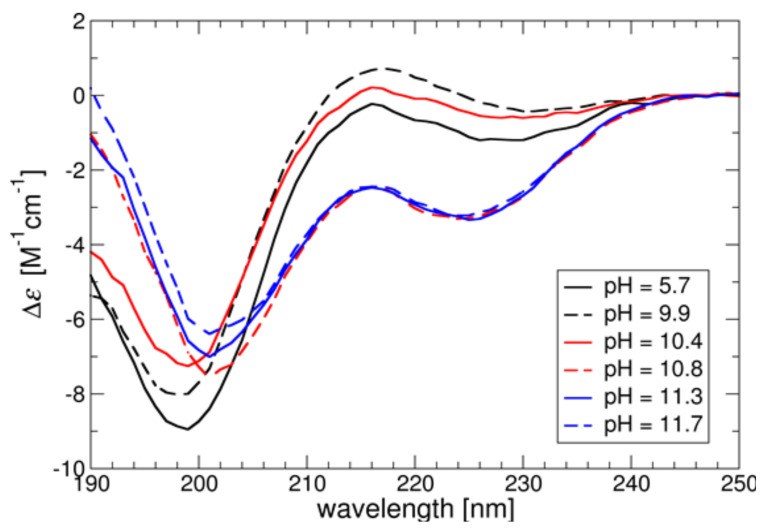
Circular dichroism spectra of P-Arg molecule at various pHs. The measurements were performed for ionic strength of 10^−2^ M NaCl at *T* = 298 K.

As can be seen from the CD curves from Figure 3, in the pH range 5.7–10.4, at which the P-Arg molecule is highly charged, the polypeptide is predominantly in a random coil configuration, with a moderate β-sheet and a very small contribution of the α-helix structure, which is in line with MD simulations. However, in the pH range 10.8–11.7 the CD spectrum shows that the polypeptide partially takes the α-helix structure (see Table 3). When the pH was increased to 12.4 the polypeptide precipitated (data not shown). These results are consistent with previously performed CD measurements for hydrobromide polyarginine [31].

The results graphically shown in Figure 3, and the resulting structural data are additionally collected in Table 3.

**Figure 4:**
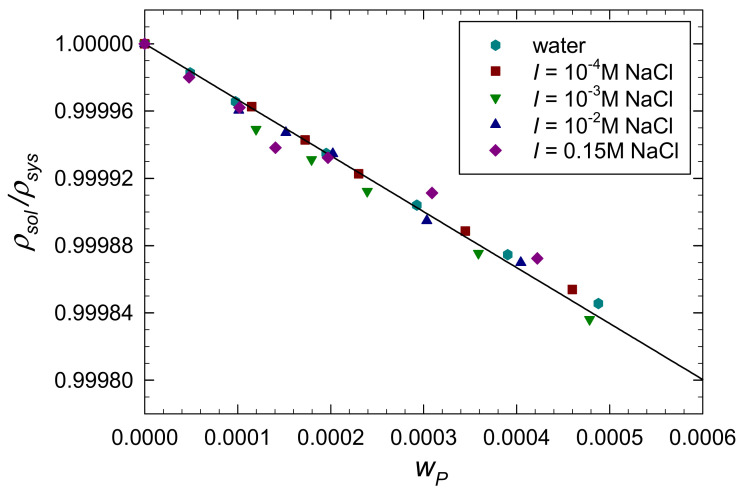
The dependence of the *ρ_sol_/ρ_sys_* on the mass concentration of P-Arg solutions *w_p_* for pure water and its solutions of an ionic strength equal to 10^−4^ to 0.15 M at pH 5.7 and *T* = 298 K. The solid line shows the linear fit of experimental data with the slope *s_p_* equal to –0.33.

Regarding physicochemical characteristics of P-Arg molecule, its density was determined by the solution dilution method as previously described [44,53]. Initially, the macroion solutions of known mass fraction *w_p_* up to 5 × 10^−4^ in NaCl and fixed concentrations equal to 10^−4^, 10^−3^, 10^−2^ and 0.15 M were prepared. The density of these solutions, denoted by *ρ_sys_*, was measured using the densitometer with a precision of 5 × 10^−3^ kg m^−3^. The dependence of *ρ_sol_*/*ρ_sys_* on *w_p_* acquired in this way (where *ρ_sol_* is the density of the pure solvent as water and pure NaCl solution of a given ionic strength) is presented in Figure 4.

As can be seen in Figure 4 the results are independent of the NaCl concentration, and they can be fitted by a straight line dependence with the slope −0.33. Therefore, the P-Arg molecule density *ρ_p_* can be calculated from the dependence [44,56,57]:

(2) $$\rho_{p}=\frac{\rho_{sol}}{1+s_{p}}$$

Using the above slope value one can calculate from Equation (2) that *ρ_p_* = 1.5 ± 0.04 × 10^3^ kg m^−3^ for both pure water and the NaCl concentration range 10^−4^ to 0.15 M. This value is only slightly lower than that derived from MD modeling.

Using this density and the average molar mass of the sample equal to 42 kg mol^−1^, one can calculate the volume of the P-Arg molecule from the formula *v_m_* = 10^27^ × *M_n_/(ρ_p_ Av)*, which is equal to 46.5 nm^3^. In addition, using the chain diameter of 0.84 nm derived from MD modeling, one can calculate the maximum extended length (*L_e_*) of the molecule, assuming its cylindrical shape, from the formula:

(3) $$L_{e}=\frac{4v_{p}}{\left(\pi d_{c}^{2}\right)}$$

An extended molecule length equal to 84 nm was calculated, corresponding to the aspect ratio parameter *λ = L_e_/d_c_* of 100.

Analogously, considering that the molar mass of the monomer is equal to 0.174 kg mol^−1^, one can predict that there are on average *N_m_* = 241 monomers in the P-Arg molecule. All these physicochemical data are given in Table 4.

**Figure 5:**
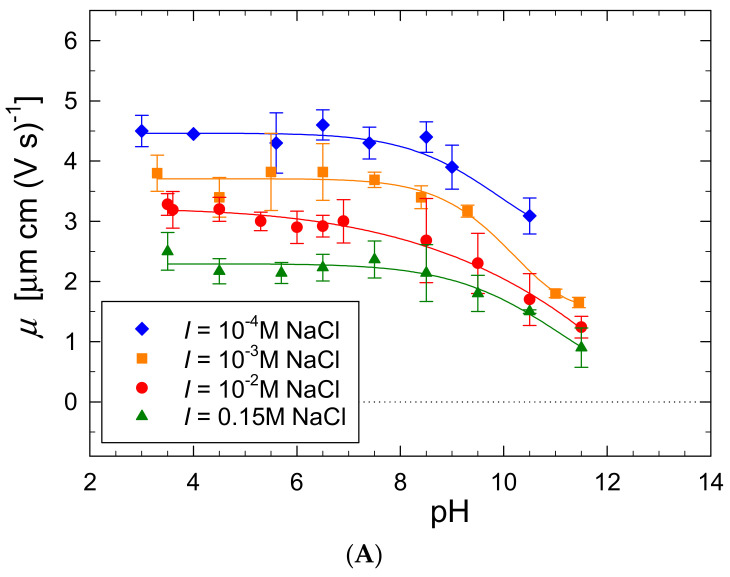

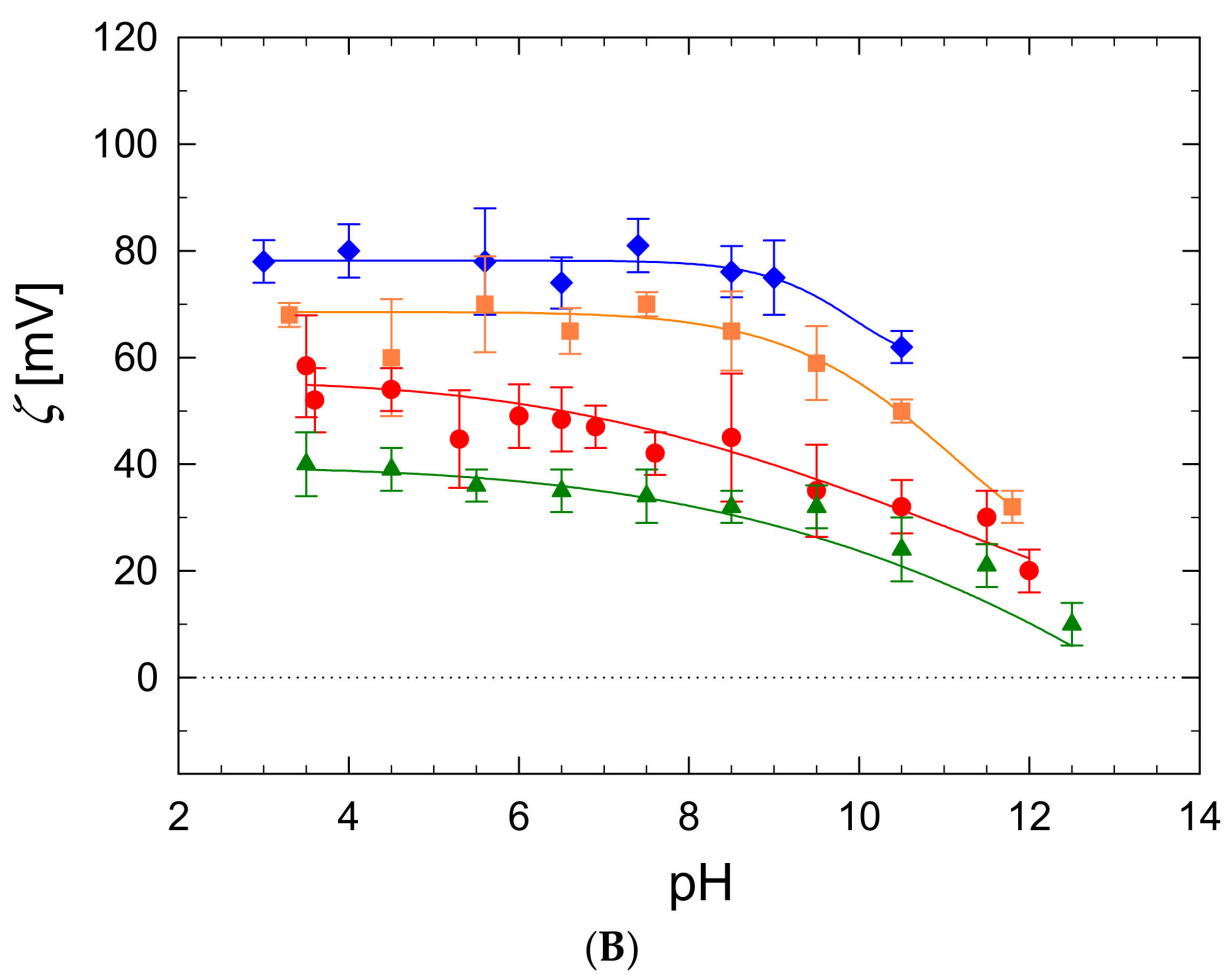
(**A**) Dependence of the electrophoretic mobility of the P-Arg molecule on pH (LDV) (**B**) Dependence of the zeta potential on pH. The measurements were performed at *T* = 298 K.

Except for the density, the most relevant physicochemical characteristics of macroions are the electrophoretic mobility *μ_e_* and the diffusion coefficient *D* that can be acquired via direct LDV and DLS measurements. The dependence of the P-Arg molecule electrophoretic mobility on pH changed within the range of 4 to 12 is shown in Figure 5 for various NaCl concentrations. One can observe that the mobility is positive for the entire pH range and monotonically decrease with the NaCl concentration. At pH 5.6 the mobility is equal to 4.5 and 2.3 μm cm (V s)^−1^ for the NaCl concentration equal to 10^−4^ and 0.15 M, respectively.

These electrophoretic mobility values correspond to the zeta potential of the molecule, calculated from the Debye–Hückel–Henry model, equal to 80 and 38 mV for ionic strength 10^−4^ and 0.15 at pH 5.8, respectively. The zeta potential slightly decreased with pH attaining 60 and 27 mV for ionic strength 10^−4^ and 0.15 at pH 10.5, respectively (see Figure 5B). The measurements confirmed that the electrokinetic charge of the P-Arg molecule is positive. Moreover, the decrease in the zeta potential under basic conditions correlates with the CD data, i.e., as long as the molecule is strongly charged the α-helical structure is not present in the P-Arg molecule conformation.

**Figure 6:**
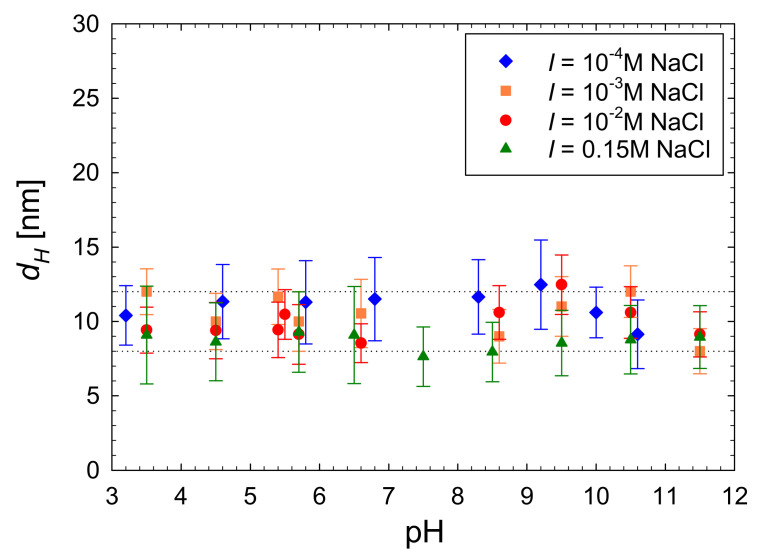
The dependence of the hydrodynamic diameter of the P-Arg molecule on pH obtained experimentally from DLS measurements. The dotted lines correspond to the average *d_H_* value equal to 12 and 8 nm for 10^−4^ M and 0.15 M NaCl, respectively. The measurements were performed at *T* = 298 K.

In an analogous way, the P-Arg molecule diffusion coefficient was acquired applying the DLS measurements at pH within the pH range 4–12 and for various NaCl concentrations. Using these primary data, the hydrodynamic diameter of the molecule *d_H_* was calculated from the Einstein–Stokes formula. It should be mentioned that in contrast to the diffusion coefficient, the hydrodynamic diameter is independent of the temperature and the solvent viscosity. The dependence of *d_H_* on pH is shown in Figure 6 for various NaCl concentrations. As can be seen, for a fixed NaCl concentration, the hydrodynamic diameter remains practically independent of pH up to 11, which suggests that there were minimal changes in the molecule conformations in accordance with the CD data. However, at a fixed pH, the hydrodynamic diameter decreased with NaCl concentration assuming the values of 12 and 8 nm for 10^−4^ and 0.15 M, respectively (pH 5.6). The decrease in the hydrodynamic diameter can be attributed to the decreased length of the molecule as previously observed for other cationic macroions [53,58].

Notably, using the electrophoretic mobility and the diffusion coefficient or the hydrodynamic diameter, one can determine the electrokinetic charge of the P-Arg molecule denoted by *q_e_* from the Lorentz–Stokes relationship [53,58].

(4) $$q_{e}=\frac{kT}{D}\mu_{e}=3\pi \eta d_{H}\mu_{e}$$

where *k* is the Boltzmann constant, *T* is the absolute temperature and η is the dynamic viscosity of the solution.

Consequently, the number of elementary charges *N*_c_ per one PLL molecule can be calculated from the formula:

(5) $$N_{c}=q_{e}/e\;$$

where *e* is the elementary charged equal to 1.602 × 10^−19^ C.

One should underline that Equation (5) is valid for an arbitrary charge distribution, the shape of molecules and the electrophoretic mobility. However, its accuracy decreases if the double-layer thickness $\kappa^{-1}={\left(\varepsilon kT/2e^{2}I\right)}^{1/2}$ (where *ε* is the electric permittivity of the solvent, *e* is the elementary charge, $I=\left(\sum c_{i}z_{i}^{2}\right)/2$ is the ionic strength, *c_i_* are the ion concentrations, *z_i_* are the ion valences) becomes smaller than the molecule hydrodynamic diameter.

Using the experimental data obtained from LDV and DLS one obtains from Equation (5) that *N*_c_ decreases at pH 5.6 from 40 (10^−4^ M NaCl) to 13 (0.15 M); see Table 5. Given that the number of monomers in the P-Arg molecule is equal to 241 one can predict that the electrokinetic charge only amounts to 17 to 4% of the nominal molecule charge, respectively. Notably, such behavior was previously reported for other macroions such as PAH [39,59], PDADMAC [53,60] and PLL [44].

### 3.3. Dynamic Viscosity Measurements

**Figure 7:**
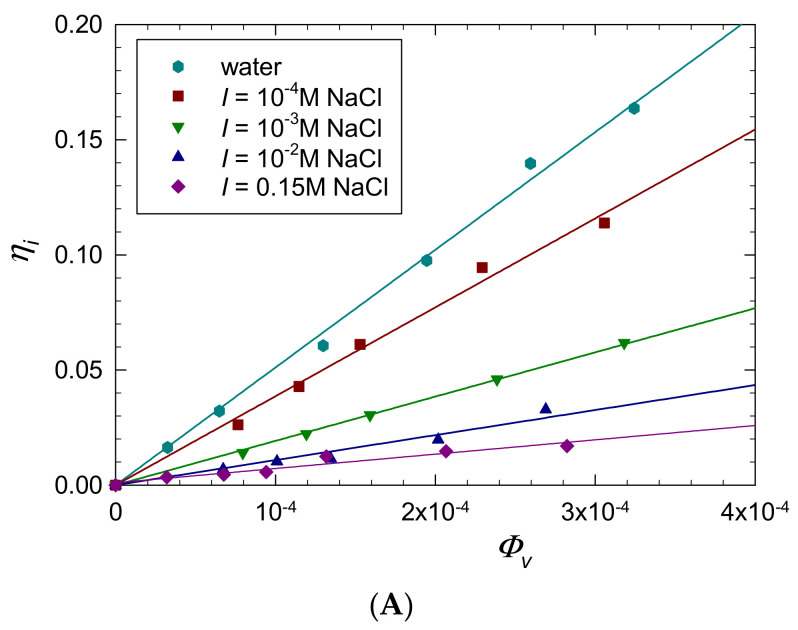

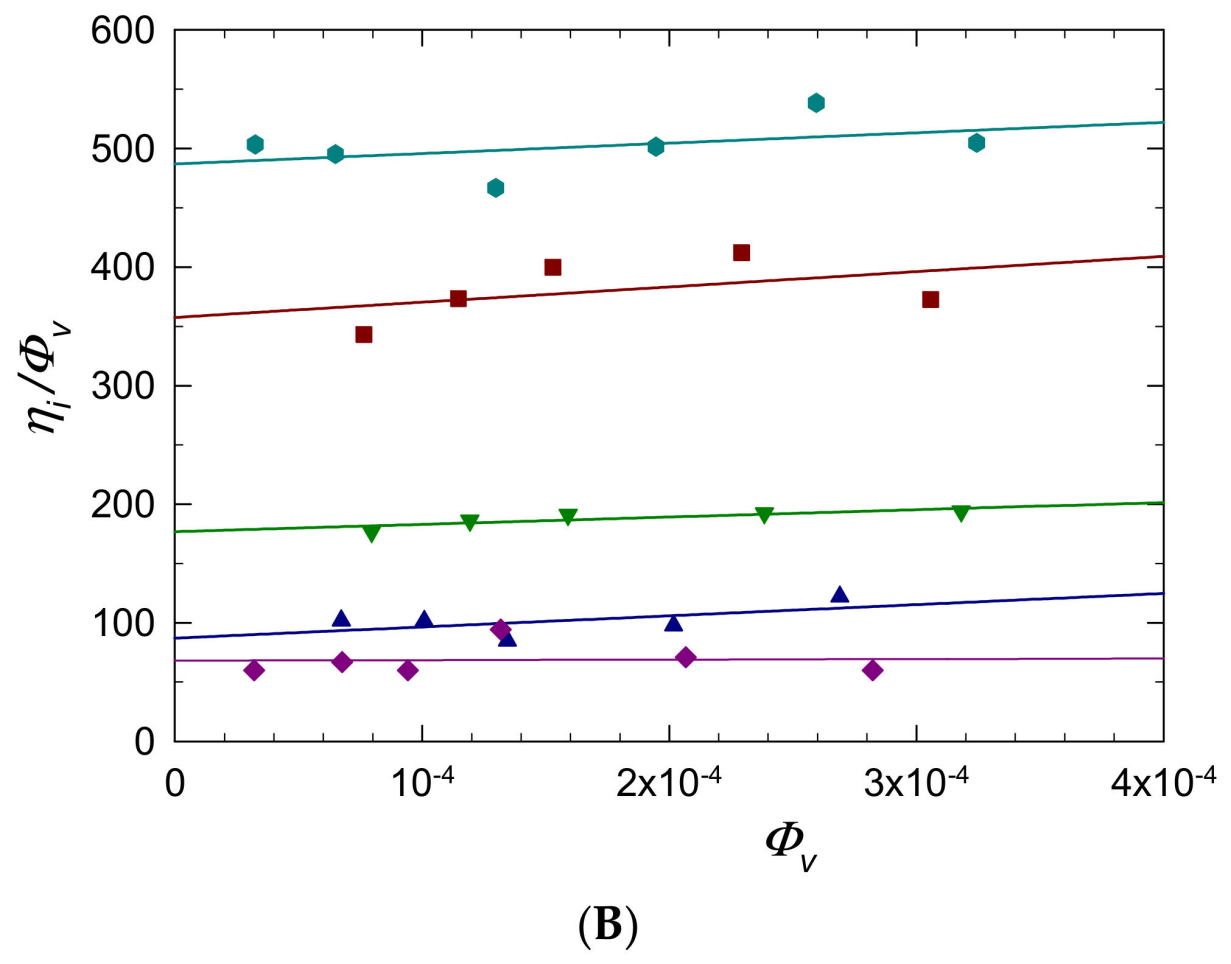
(**A**) Dependence of the relative viscosity *η_i_ = η_p_*/*η_e_* on the volume concentration of P-Arg solutions *Φ*_V_. The solid line denotes a linear fit of experimental data. (**B**) Dependence of *η_i_*/*Φ*_V_ on the volume concentration of P-Arg solutions *Φ*_V_. The solid lines denote the linear fits of experimental data.

**Figure 8:**
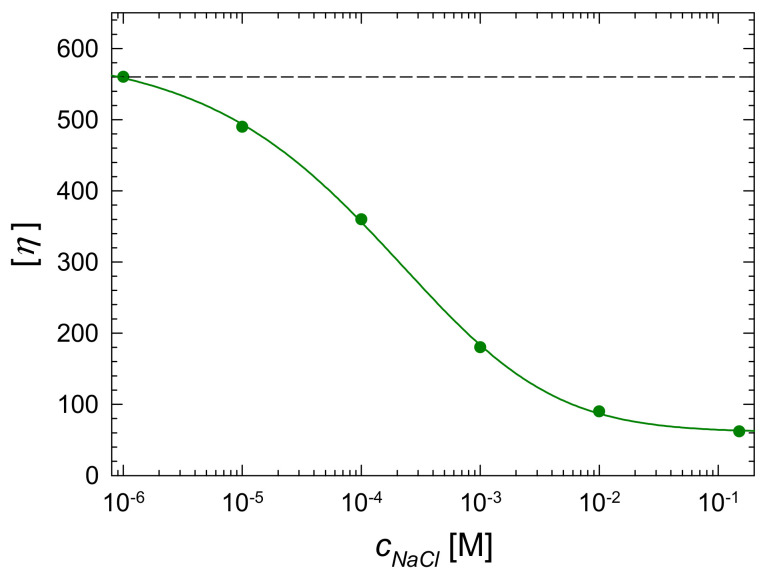
The dependence of the intrinsic viscosity [*η*] of P-Arg on the NaCl concentration. The points show the experimental data, the dashed lines show the theoretical slender body limit for fully extended molecules predicted from MD modeling and the solid lines denote the nonlinear fit of experimental data.

Initially, in order to determine the intrinsic viscosity of P-Arg molecule, the dependencies of the dynamic viscosity of its solutions *η_p_* on the volume fraction *Φ*_V_ *= c_b_*/*ρ_p_* were determined for various NaCl concentrations in the range of 2 × 10^−5^ (distilled water) to 0.15 M (where *c_b_* is the macroion mass concentration in the solution). These primary results were converted to the dependencies of the relative viscosity *η_i_ = η_p_*/*η_e_* (where *η_e_* is the pure electrolyte viscosity) on the P-Arg volume fraction and are shown in Figure 7A. The slopes of these dependencies in the limit of low volume fraction correspond to the intrinsic viscosity [*η*] of the P-Arg molecule for a given NaCl concentration. The precision of this method of the intrinsic viscosity determination can be increased by plotting *η_i_*/*Φ*_V_ against *Φ*_V_ (Figure 7B). The experimental values of the intrinsic viscosity obtained in this way for various NaCl concentrations are shown in Figure 7 and Figure 8 and in Table 6.

One can infer from the data shown in Table 6 that the intrinsic viscosity for 10^−5^ M NaCl is equal to 490 ± 10, and the extrapolated value for 10^−6^ M NaCl is equal to 560, which is assumed to approach the limiting value for fully extended chain. It is also worth mentioning that for all NaCl concentrations up to 0.15 M, the intrinsic viscosity is equal to 62, which significantly exceeds the Einstein results for spherically shaped macromolecules where [*η*] = 2.5. This behavior suggests that P-Arg molecules assume extended conformation for the entire NaCl concentration range, which corresponds to the slender body hydrodynamic regime [61]. Therefore, the experimental data can be quantitatively interpreted using the theoretical results pertinent to the zero shear rate intrinsic viscosity for slender bodies [61]:

(6) $$\left[\eta ]=c_{1}{}_{v}\frac{\lambda^{2}}{\ln2\lambda -0.5}+c_{2v}\frac{\lambda^{2}}{\ln2\lambda -1.5}+c_{v}=f_{v}([\lambda ]\right)$$

where *c*_1v_ = 3/15, *c*_2v_ = 1/15 and *c*_v_ is equal to 14/15 for blunt cylinders and *λ = L_e_/d_c_* is the molecule axis ratio assumed to be much larger than unity.

Therefore, knowing the intrinsic viscosity, the aspect ratio parameter for an equivalent cylinder can be calculated by a numerical inversion of Equation (6)

(7) $$\lambda =f_{v}^{-1}([\eta ])$$

where $f_{v}^{-1}([\eta ])$ is the inverse function of $f_{v}([\eta ])$.

One can also calculate *λ* using the iterative scheme, which produces the following formula [44]:

(8) $$\lambda ={⟮\frac{15_{}^{}{\left[\eta \right]}_{}}{\frac{3}{\left(\ln2\lambda_{1}-0.5\right)}+\frac{1}{\left(\ln2\lambda_{1}-1.5\right)}}⟯}^{\frac{1}{2}}$$

where

(9) $$\lambda_{1}={\left(15_{}^{}[\eta ]\right)}^{\frac{1}{2}}$$

The precision of Equation (8) is ca. 1% for the intrinsic viscosity exceeding 50.

One can thus calculate that the *λ* parameter for P-Arg molecules is equal to 89 and 97 for NaCl concentrations of 10^−5^ and 10^−6^ M, respectively. For the NaCl concentration of 0.15 M, the *λ* parameter significantly decreases and assumes the value of 28. 

Exploiting the large precision of the *λ* parameter determination via the dynamic viscosity measurements one can calculate several derivative parameters characterizing the for P-Arg molecules in dilute electrolyte solutions, which are more time-consuming and impractical to measure. Primarily, one can determine the molar mass of a P-Arg sample from the linear dependence:

(10) $$M_{m}=\frac{M_{1}d_{c}}{l_{m}}\lambda =C_{m}{}_{}^{}\;\lambda$$

where the $C_{m}{}_{}^{}$ constant is equal to 0.439 for $d_{c}$ = 0.84 and *l_m_* = 0.333 nm, respectively.

Taking the limiting value of *λ* = 97 derived from the viscosity measurements, one obtains from Equation (10) that the average molar mass of our sample was equal 43 kg mol^−1^, which agrees within the error bounds with the value given by the manufacturer. On the other hand, for *λ* = 75 and *d_c_* = 0.93(10^−4^ NaCl), where *Cm =* 0.493 one obtains from Equation (10) $M_{m}$ = 37 kg mol^−1^.

Analogously, the molecule hydrodynamic diameter can be calculated from the formula [61]:

(11) $$d_{H}=\frac{\lambda }{\left(\ln2\lambda -0.11\right)}d_{c}$$

As a consequence, the diffusion coefficient of the molecule is given by:

(12) $$D=\frac{kT}{3\pi \eta_{e}d_{H}}$$

In addition, the radius of gyration of the P-Arg molecule in the slender body limit can be calculated from the dependence:

(13) $$R_{g}=\frac{d_{c}}{12^{1/2}}\lambda$$

On the other hand, the sedimentation coefficient defined as the ratio of the hydrodynamic diameter of the molecule to the diameter of an equivalent sphere with the same volume [62] is given by:

(14) $$S_{c}=\frac{d_{H}}{d_{s}}={⟮\frac{\pi Av\rho_{p}d_{c}^{3}}{6M_{m}}⟯}^{\frac{1}{3}}\frac{\lambda }{\left(\ln2\lambda -0.11\right)}{=}_{}{⟮\frac{\pi Av\rho_{p}l_{m}d_{c}^{2}}{6M_{1}}⟯}^{\frac{1}{3}}\frac{\lambda^{\frac{2}{3}}}{\left(\ln2\lambda -0.11\right)}$$

It should be mentioned that Equation (14) is particularly useful for the low ionic strength limit because the measurements of the sedimentation coefficient using the ultracentrifuge method are rather impractical due to the electrostatic interactions among sedimentation molecules. However, in all these expressions the parameter should be calculated from Equation (7) or by a numerical inversion of Equation (8) if a larger precision is needed.

**Figure 9:**
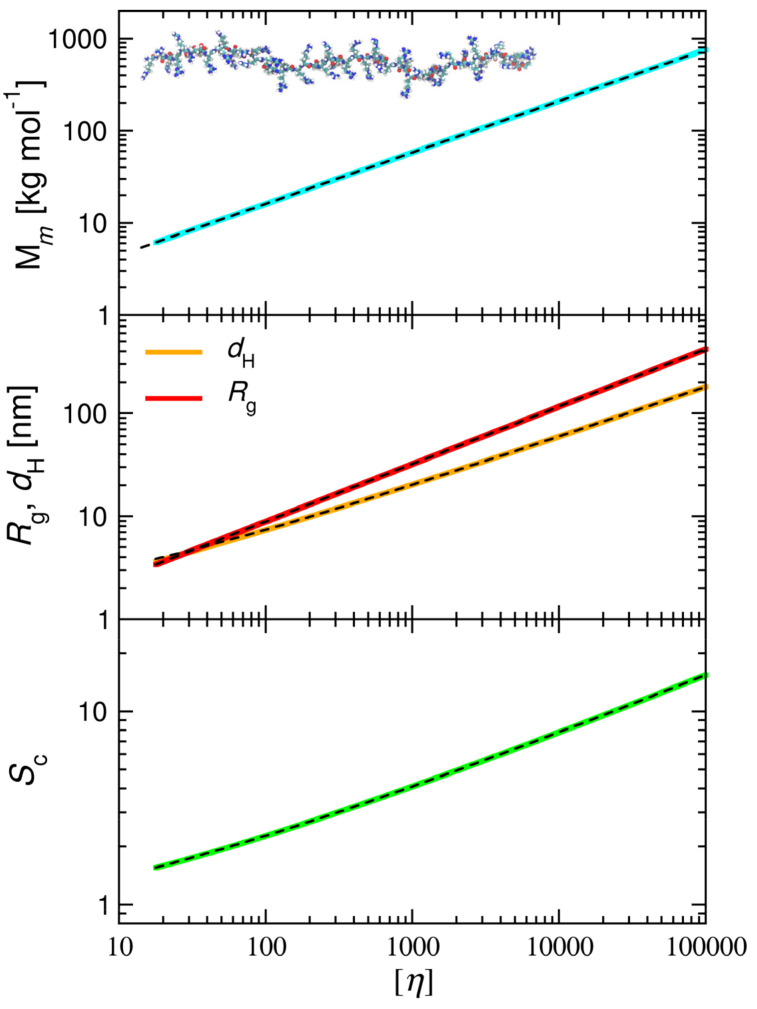
The molar mass (*M*_m_), the radius of gyration (*R*_g_), the hydrodynamic diameter (*d_H_*) and the sedimentation coefficient (*S*_c_) of P-Arg solutions vs. the intrinsic viscosity ([*η*]) in the limit of low ionic strength; theoretical results calculated from Equations (10), (11), (13) and (14). The dashed lines present fits to Equations (16)–(19). The insets show the P-Arg molecule conformation derived from MD modeling.

The dependencies of the molar mass, the hydrodynamic diameter, the radius of gyration and the sedimentation coefficient of P-Arg calculated using the above formulae are plotted in Figure 9. Useful interpolation functions for the molar mass and the radius of gyration are as follows:

(15) $$\lambda =2.81{\left[\eta \right]}^{0.558}$$

(16) $$M_{m}=1.23{\left[\eta \right]}^{0.558}$$

(17) $$R_{g}=0.681_{}^{}{\left[\eta \right]}^{0.558}$$

where [*η*] is the intrinsic viscosity in the limit of low electrolyte concentration (dimensionless parameter), *M_m_* is expressed in [kg mol^−1^], and *R_g_* in [nm].

Analogously, the hydrodynamic diameter and the sedimentation coefficient are interpolated by the functions:

(18) $$d_{H}=1.18+0.659{\left[\eta \right]}^{0.487}$$

(19) $$S_{c}=0.543+0.412{\left[\eta \right]}^{0.312}$$

with *d_H_* expressed in [nm] and Sc is a dimensionless variable.

## 4. Conclusions

Poly-L-arginine (P-Arg) molecule conformations and basic physicochemical properties in the NaCl electrolyte were thoroughly determined using molecular dynamics (MD) modeling, CD, LDV, DLS and dynamic viscosity measurements. The modeling was used to calculate the molecule density, the monomer length and the equivalent cylinder diameter for a fully extended chain.

These theoretical results enabled quantitative interpretation of experimental data comprising the electrophoretic mobility, the diffusion coefficient, the hydrodynamic diameter and intrinsic viscosity of molecules determined for the ionic strength range 10^−5^ to 0.15 M. It is shown inter alia that the molecule exhibits a positive electrokinetic charge for pH up to 12, which is, however, considerably lower than the nominal charge calculated from ionization equilibrium. It is also shown that there is no secondary structure change, especially the α-helix content for pH up to 10.4.

From the dynamic viscosity measurements, it is deduced that the molecules assume extended conformations at all ionic strengths, which corresponds to the slender body hydrodynamic limit. These measurements also yielded the length to chain diameter parameter of the molecule, which can be used to precisely determine the molar mass of any P-Arg sample. The proposed method, only requiring the intrinsic viscosity of the macroion solution in the limit of low ionic strength (distilled water), may prove superior to others such as MALS and size exclusion chromatography, which are rather inadequate for cationic macroions.

Except for the molar mass, the described method can be used to predict the diffusion coefficient, the hydrodynamic diameter, the radius of gyration and the sedimentation coefficients of macroion molecules.

One can also expect that the acquired results can be exploited for controlling P-Arg molecule adsorption at solid/liquid interfaces, which is often applied for producing supporting layers for nanoparticle and protein immobilization or for macroion shell formation of nanocapsules used for targeted drug delivery.

## Figures and Tables

**Table 1:** Physicochemical characteristics of P-Arg molecule derived from MD modeling, *T* = 298 K.

Quantity (Unit), Symbol	Value	Remarks
Chemical structure of monomer	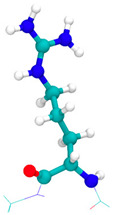	Monomer molar mass, M_1_ = 0.174 kg mol^−1^
Density (kg m^−3^), *ρ_p_*	1.58 ± 0.03 × 10^3^	This work, MD modeling
Monomer volume (nm^3^), *ν_1_*	0.183 ± 0.002	Calculated as M_1_/(*Av ρ*_p_)
Monomer length for the extended chain (nm), *l_m_*	0.333 ± 0.001	This work, MD modeling

**Table 2:** The average and the maximum EtE length of the P-Arg molecule composed of 25, 30, 35, 40, 45 and 50 monomers derived from MD modeling.

Quantity (Unit), Symbol	Number of Monomers, *N_m_*					
	25	30	35	40	45	50
Average end-to-end distance (nm)	5.95 ± 0.01	6.42 ± 0.01	8.17 ± 0.01	8.93 ± 0.01	10.24 ± 0.01	10.78 ± 0.01
Maximum end-to-end distance (nm) (contour length)	8.38 ± 0.01	9.89 ± 0.01	11.48 ± 0.01	13.24 ± 0.01	15.16 ± 0.01	16.77 ± 0.01
Molecule volume (nm^3^), *v_m_*	4.58 ± 0.05	5.49 ± 0.06	6.41 ± 0.7	7.32 ± 0.08	8.24 ± 0.09	9.15 ± 0.10
Chain diameter (nm), *d_c_* (*I* = 10^−3^ M)	1.00 ± 0.02	1.04 ± 0.02	1.00 ± 0.02	1.02 ± 0.02	1.01 ± 0.02	1.04 ± 0.01
Diameter of extended chain (nm) (*I* = 0 M)	0.83 ± 0.02	0.84 ± 0.02	0.84 ± 0.02	0.84 ± 0.02	0.83 ± 0.02	0.83 ± 0.02

**Table 3:** The percentage of the secondary structure contents for P-Arg at different pH at *I* = 10^−2^ M NaCl and *T* = 298 K.

pH	α-Helix	β-Sheet	Turn	Others	NRMSD
5.7	1	31	18	50	0.0393
9.9	0	33	16	51	0.0454
10.4	0	29	18	53	0.038
10.8	13	23	13	51	0.0507
11.3	15	21	13	51	0.052

NRMSD is normalized root mean square deviation.

**Table 4:** Physicochemical characteristics of P-Arg used in experimental studies, *T* = 298 K, pH 5.4–5.8.

Quantity (Unit), Symbol	Value	Remarks
Average molar mass (kg mol^−1^), *M_n_*	42	Manufacturer, viscosity method
Density (kg m^−3^), *ρ_p_*	1.5 ± 0.04 × 10^3^	This work, dilution method
Average number of monomers in the molecule, ${\bar{N}}_{m}$	241 ± 10	Calculated as *M_n_*/*M*_1_
Average molecule volume (nm^3^), *ν_p_*	46.5	Calculated as 10^27^ × *M_n_*/(*ρ_p_ Av*)
Equivalent sphere diameter (nm)	4.73	Calculated as: (6 *ν_p_*/π)^1/3^
Extended length (maximum) (nm), $\bar{L_{e}}$	84	Predicted from the cylinder (rod) model: 4 *ν_p_*/(*π d_c_*^2^)
	80	Calculated as: ${\bar{N}}_{m}$ *l_m_*
Average aspect ratio parameter, *λ*	100	Calculated from the cylinder (rod) model, $\bar{L_{e}}$/*d*_c_
	96	Predicted from MD calculations

Assumed *d_c_* = 0.84 nm, *l_m_* = 0.333 nm.

**Table 5:** Physicochemical characteristics of P-Arg used in experimental studies, *T* = 298 K, pH 5.4–5.8.

*I* (M)	*κ*^−1^ (nm)	*D* (m^2^ s^−1^)	*d_H_* (nm)	*μ_e_* (μm cm (V s)^−1^)	*ζ* (mV)	*N_c_*	α*
1 × 10^−5^	68.7	2.9 ± 0.4 × 10^−11^	17 ± 5	4.7 ± 0.25	85	63	0.26
10^−4^	30.5	4.1 ± 0.4 × 10^−11^	12 ± 3	4.2 ± 0.25	78	40	0.17
10^−3^	9.63	4.5 ± 0.4 × 10^−11^	11 ± 3	3.7 ± 0.25	70	32	0.13
10^−2^	3.05	4.9 ± 0.3 × 10^−11^	10 ± 2	3.2 ± 0.2	55	25	0.10
0.15	0.786	6.1 ± 0.3 × 10^−11^	8 ± 3	2.1 ± 0.2	38	13	0.04

α* = N_c_/N_mx_ is the effective ionization degree; $d_{H}=kT/3\pi \eta D$ is the hydrodynamic diameter; $N_{c}=\frac{kT}{De}\mu_{e}$ is the number of uncompensated charges.

**Table 6:** The intrinsic viscosity [*η*], the *λ = L_e_/d_c_* parameter, the equivalent cylinder chain diameter *d*_c_, the cylinder lengths *L*_c_ of the P-Arg molecules for various ionic strengths derived from viscosity and DLS measurements (pH 5.6).

*c_NaCl_* [M]	*[η]* [1]	*λ* [1]	*L_c_* [nm]	*d_c_* [nm]
10^−6^	560 *	97 *	81 *	0.84 0.84 ± 0.02 **
10^−5^	490 ± 10	89 ± 3	79 ± 3	0.87 ± 0.02
10^−4^	360 ± 10	75 ± 3	70 ± 3	0.93 ± 0.02
10^−3^	180 ± 10	51 ± 3	54 ± 2	1.06 ± 0.03 1.03 ± 0.02 **
10^−2^	90 ± 5	34 ± 2	41 ± 2	1.2 ± 0.05
0.15	62 ± 5	28 ± 2	36 ± 2	1.3 ± 0.05

* extrapolated value from the dynamic viscosity measurements, ** calculated from the MD modeling.

## Supplementary Materials

The following supporting information can be downloaded at: https://www.mdpi.com/article/10.3390/ijerph19063588/s1, Figure S1: Determining density of poly-L-arginine using Molecular Dynamic Modeling.
