# Supplementary material for: Poly-L-Arginine Molecule Properties in Simple Electrolytes: Molecular Dynamic Modeling and Experiments
Source: Int J Environ Res Public Health. 2022 Mar 17;19(6):3588. doi: 10.3390/ijerph19063588 (PMC8951092; doi:10.3390/ijerph19063588)

**Figure S1.** Determining density of poly-L-arginine using Molecular Dynamic Modeling.

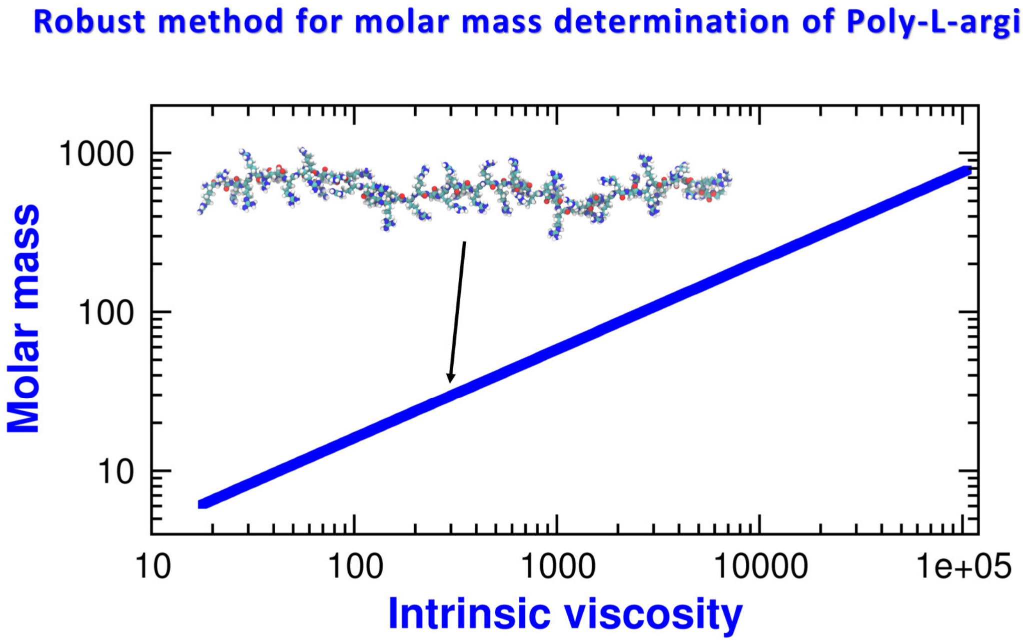

Supplement: Supplementary file 1 [file ijerph-19-03588-s001.zip › ijerph-1588706-supplementary.pdf]
